# Complete chloroplast genome structural and phylogenetic analysis of *Physostegia virginiana* (L.) Benth. 1930 (Lamiaceae)

**DOI:** 10.1080/23802359.2025.2528568

**Published:** 2025-07-16

**Authors:** Changmei Du, Tingting Xiong, Yan Dong, Haishuo Gao, Yan Zhang, Jianhua Yue

**Affiliations:** ^a^ School of Horticulture, Xinyang Agriculture and Forestry University, Xinyang, PR China; ^b^ School of Forestry, Xinyang Agriculture and Forestry University, Xinyang, PR China; ^c^ Xinyang Camellia oleifera Industry Development Center, Xinyang, PR China; ^d^ Department of Plant Science and Technology, Shanghai Vocational College of Agriculture and Forestry, Shanghai, PR China

**Keywords:** chloroplast genome, *Physostegia virginiana* (L.) Benth., Lamiaceae, phylogenetic relationship

## Abstract

*Physostegia virginiana* (L.) Benth. (1930) is an ornamental plant with great potential for landscaping application and cut-flower using in China. To explore its phylogenetic position and analyze the characteristics of its chloroplast (cp) genome, this study sequenced the whole cp genome of *P. virginiana*. The results revealed that the total length of the cp genome was 152,880 bp, and the lengths of the large single-copy region (LSC) and small single-copy region (SSC) were 84,428 and 17,396 bp, respectively. The lengths of the inverted repeat regions (IRa and IRb) were both 25,528 bp. The cp genome of *P. virginiana* contains 131 coding genes, namely, 86 protein-coding genes, 37 tRNA genes and eight rRNA genes. The total GC content of the cp genome was 38.38%. The phylogenetic results revealed that *P. virginiana* was closely related to *Gomphostemma lucidum*. The results of this study provide a molecular basis for the phylogenetic localization of *P. virginiana* and basic data for its identification and resource development.

## Introduction

*Physostegia virginiana* (L.) Benth. (1930) also known as false dragon head flower, is a perennial persistent herbaceous flower belonging to the genus *Physostegia* in the Lamiaceae family. It is native to Eastern North America and grows in moist to mesic prairies, the borders of woodlands and riverbanks (Gudžinskas 2017). *P. virginiana* is famous for its long flowering period, gorgeous color and unique flower shape. The flowering period is from July to October. The pinkish-purple spikes, which are 30 cm long, and the flower tubes, which are 2.5 cm long, are particularly bright color. These are also important characteristics that attract specific pollinators such as bees and butterflies. It exhibits strong adaptability, being cold-resistant, drought-resistant, and tolerant of high soil fertility. It can grow in places where many other species cannot survive, making it an important ornamental plant in many public gardens around the world (Wang et al. 2021). In China, in addition to being used as a landscape plant in parks and wetlands, it is also a very popular cut-flower plant (Dong et al. 2023). *P. virginiana* can be developed and utilized as an important flower-viewing plant resource in summer landscapes. The position of *P. virginiana* in the plant classification system is relatively stable. In the classification systems such as angiosperm classification system and APG lll (2009), it has always been classified under the genus *Physostegia* of the family Lamiaceae. However, the specific taxonomic position of *P. virginiana* within the Lamiaceae family remains unclear, and there are certain research gaps. Its complete chloroplast (cp) genome sequence provides valuable resources for the phylogenetic analysis. Moreover, it enables the identification of important genes related to photosynthesis, growth and development, and stress resistance, which is of great significance for studying the genetic evolution of *P. virginiana*.

In this study, the cp genome of *P. virginiana* was sequenced, assembled, and annotated. The structural characteristics of the cp genome were analyzed in detail. The phylogenetic relationships of plants in the genus were explored, filling the gap in the phylogenetic research of plants in the genus *Physostegia* and providing a scientific reference for the genetic diversity and phylogenetic evolution of plants in this genus.

## Materials and methods

**Figure 1.**
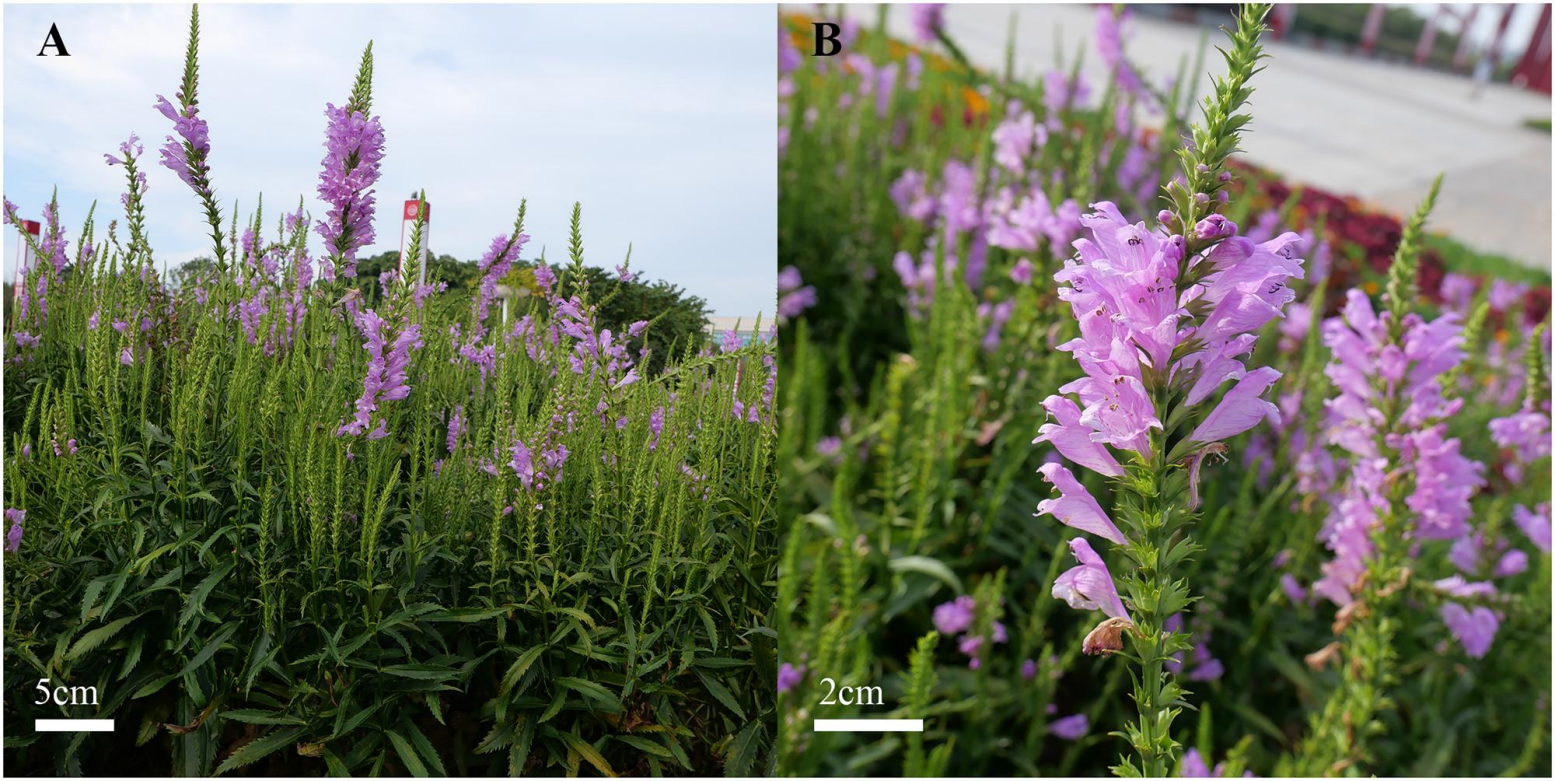
Plant morphological characteristics (A) and floral organ morphology (B) of *P. virginiana*. The photos of *P. virginiana* were taken by Changmei Du at Baihua Garden, Xinyang, Henan, China. There are no copyright issues. Morphological characteristics: Stems clustered and erect, quadrilateral, leaves opposite, lanceolate, bright green, margin serrated. Terminal spike, pinkish-purple.

The samples of *P. virginiana* were collected from Baihua Garden (114° 12′ E, 32° 16′ N, altitude: 102 m; Figure 1) in Xinyang City, Henan Province, China. Professor Jianhua Yue from Xinyang Agriculture and Forestry University identified the sample information. The specimen was deposited at the Herbarium of the Horticultural Plant Biotechnology Laboratory, Xinyang Agriculture and Forestry University (contact Changmei Du, Yanxuedcm@163.com), and the voucher code is PVB20231120. Fresh samples were sent to Shanghai Origingene Biotechnology Co., Ltd., for complete genome sequencing.

### Genomic DNA extraction and sequencing

The total DNA was extracted using the Plant Genomic DNA Extraction Kit (Magen), and the sequencing library was subsequently constructed by terminal repair, A-tail addition, sequencing splice addition, purification, PCR amplification and other methods. The Illumina NovaSeq 6000 (Illumina Inc., San Diego, CA) platform was used for sequencing, and a total of 8.9 Gb of raw sequence data was uploaded to the NCBI Data Center (http://www.ncbi.nlm.nih.gov/Traces/sra).

### Chloroplast genome sequence assembly and annotation

Getorganelle v.1.7.0 (Jin et al. 2020) was used to assemble the obtained clean reads. The cp genome annotation software programs PGA (Qu et al. 2019) and Geseq (Tillich et al. 2017) were used for gene annotation of the spliced cp genome, and the annotation results of all the samples were manually corrected. BWA (v.0.7.17-r1188) was used to process comparison data, SAMtools (v.1.9) was used to calculate coverage depth, and then visualized using ggplot2 in R (Li and Durbin 2009).

The cp genome sequence and gene annotation information have been uploaded to the NCBI database, and the GenBank no. is OR832244.2. Subsequently, CPGview (http://www.1kmpg.cn/cpgview/; Liu et al. 2023) was used to generate a circular genome map.

### Phylogenetic analysis

To investigate the phylogenetic placement of *P. virginiana*, the complete cp genome sequences of 26 species, namely, 23 species of Lamiaceae, one species of Verbenaceae and two species of Gesneriaceae, were obtained from the GenBank database. Multiple sequence comparisons were performed using MAFFT software (Katoh and Standley 2013). The phylogenetic trees were constructed by maximum likelihood (ML) method and Bayesian method. RAxML software (Stamatakis 2014) was used to build a ML tree with bootstrap method based on GTRCATI model and the bootstrap value was set to 1000. The GTR+I + Γ model was set up using MrBayes v3.2.6 for analysis under Bayesian inference (Ronquist et al. 2012). Nine species of the Lamiaceae family were selected for boundary analysis using CPJSdraw v1.0.0 (Li et al. 2023). Then, the cp genomes of these species were compared using the DnaSP 6.0 (Rozas et al. 2017) and mVista (Frazer et al. 2004) software.

## Results

**Figure 2.**
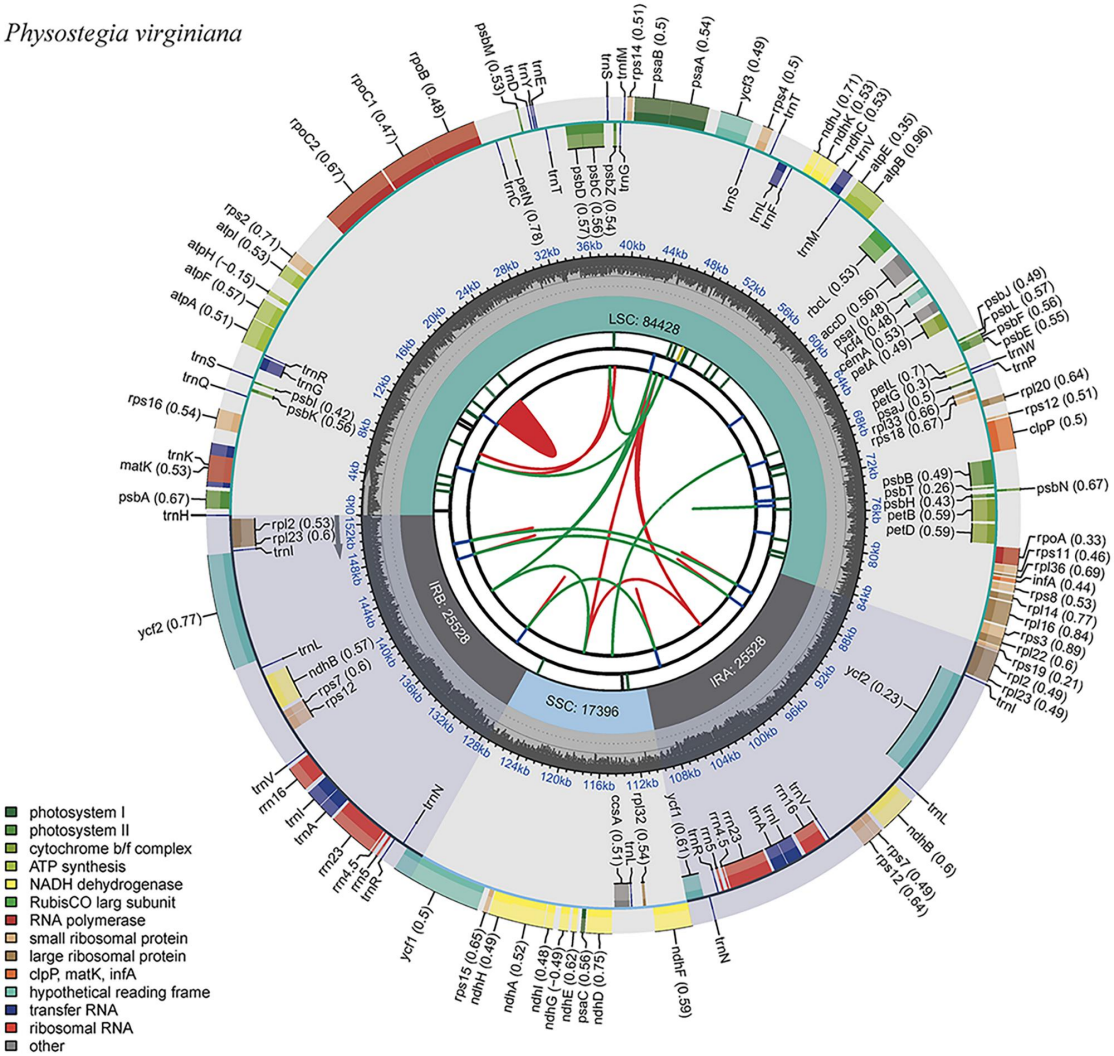
The chloroplast genome map of *P. virginiana*. The circular map of the chloroplast genome was generated using CPGview (http://www.1kmpg.cn/cpgview/). Genes are color-coded by their functional classification. The transcription directions for the inner and outer genes are clockwise and anticlockwise, respectively. The functional classification of the genes is shown in the bottom left corner.

The results of the sequencing of the cp genome of *P. virginiana* are shown in Figure 2. It is a circular double-stranded molecule with a total length of 152,880 bp and a typical tetrad structure, including a large single-copy region (LSC), a small single-copy region (SSC), and a pair of inverted repeat regions (IRs), with sizes of 84,428, 17,396, and 25,528 bp, respectively. The GC content and AT content of *P. virginiana* were 38.38% and 61.62%, respectively, indicating obvious AT bias. A total of 131 genes were annotated in the cp genome of *P. virginiana*, of which 86 were protein-coding genes, 37 were tRNA genes and eight were rRNA genes. It was also identified 13 cis-splicing genes and 2 trans-splicing genes (Figure S1). These genes can be divided into four categories according to their functions: self-replicating genes, photosynthesis-related genes, other genes and genes with unknown functions. The sequencing depth distribution of cp genome is shown in Figure S2, with an average coverage depth of 5134×.

**Figure 3.**
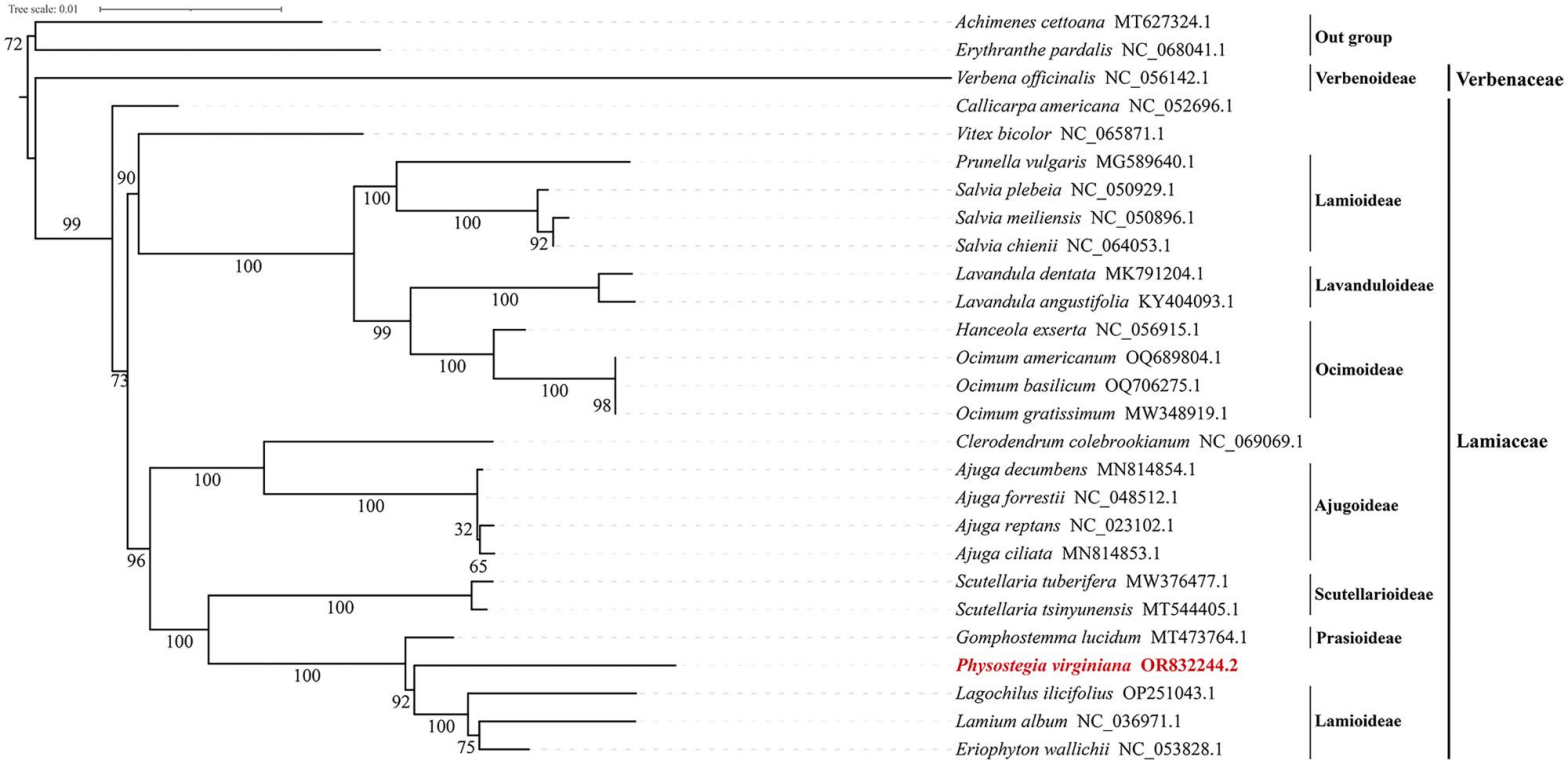
The phylogenetic tree of *P. virginiana* based on concatenated complete chloroplast genome sequence of 26 species. The bootstrap values are shown on the nodes, and the species and GenBank accession number are shown at the end of each branch. *P. virginiana* is highlighted in bold. GenBank accession numbers of the following sequences were used: *Ajuga reptans* NC_023102.1 (Khan et al. 2019), *Ajuga forrestii* NC_048512.1 (Tao et al. 2019), *Ajuga ciliata* MN814853.1, *Ajuga decumbens* MN814854.1, *Prunella vulgaris* MG589640.1 (Han and Zheng 2018), *Salvia plebeia* NC_050929.1 (Cui et al. 2020), *Salvia chienii* NC_064053.1, *Salvia meiliensis* NC_050896.1 (Su et al. 2022), *Eriophyton wallichii* NC_053828.1, *Lamium album* NC_036971.1 (Khan et al. 2019), *Lagochilus ilicifolius* OP251043.1 (Hou et al. 2024), *Gomphostemma lucidum* MT473764.1, *Scutellaria tsinyunensis* MT544405.1, *Scutellaria tuberifera* MW376477.1 (Shan et al. 2021), *Lavandula dentata* MK791204.1 (Li et al. 2019a), *Lavandula angustifolia* KY 404093.1 (Li et al. 2019b), *Hanceola exserta* NC_056915.1 (Zhu et al. 2023), *Ocimum basilicum* OQ706275.1 (Kirankumar et al. 2023), *Ocimum americanum* OQ689804.1 (Vineesh et al. 2023), *Ocimum gratissimum* MW348919.1 (Balaji et al. 2021), *Clerodendrum colebrookianum* NC_069069.1 (Fu et al. 2023), *Callicarpa americana* NC_052696. 1 (Zhao et al. 2020), *Vitex bicolor* NC_065871.1 (Gentallan et al. 2023), *Verbena officinalis* NC_056142.1 (Yue et al. 2021), *Achimenes cettoana* MT627324.1 (Li et al. 2021), *Erythranthe pardalis* NC_068041.1 (Zhao et al. 2023). Among them, *Achimenes cettoana* and *Erythranthe pardalis* are the outgroup.

The topologies of the two phylogenetic trees constructed using ML (Figure 3) and Bayesian (Figure S3) methods are highly consistent with each other. The analysis of the phylogenetic relationships among *P. virginiana* and Lamiaceae plants showed that 23 species of the Lamiaceae family were respectively clustered into two large branches. A total of 10 species from Lamioideae, Lavanduloideae, and Ocimoideae, together with *V. bicolor*, clustered into one large branch. A total of 10 species from Ajugoideae, Scutellarioideae, Prasioideae, and Lamioideae, along with *Clerodendrum colebrookianum* and *P. virginiana*, clustered into another large branch. Among them, *P. virginiana* clustered together with *Lagochilus ilicifolius*, *Lamium album*, *Eriophyton wallichii* of Lamioideae and *Gomphostemma lucidum* of Prasioideae to form a small clade. The support value for the clustering of *P. virginiana* and *G. lucidum* is 100%, and the support value for the clustering of *P. virginiana* and the species of Lamioideae is 92%.

Boundary analysis indicated that there was a high degree of similarity among species. In the four boundary regions, the positions and lengths of genes such as *rps19*, *ycf1*, *ndhF*, and *rp12* showed relatively small differences, but there were also expansions and contractions to varying degrees (Figure S4). Sequence comparison analysis showed that the sequences of each species generally had high similarity. However, there were fluctuations at the positions of genes such as *rps16*, *psbI*, *trnE-UUC*, *petA*, *rpl16*, *trnL-UAC*, and *ycf1*, which suggested that there had been variations or differentiations during the evolutionary process (Figure S5). The results of nucleotide polymorphism analysis revealed that the regions with higher PI values were located in genes such as *ycf1*, *ndhF*, *rpl32*, *rps16*, *psbI*, *trnE*, *trnT*, *trnL*, *petA-psbJ*, *rpoA*, and *rpl16*, and the PI values of other regions were all less than 0.04 (Figure S6). The above analysis results were consistent, indicating that the cp genome structures of Lamiaceae species had a high degree of conservativeness, and these differentially expressed genes could provide a basis for the identification of important genes and the screening of molecular markers in Lamiaceae plants.

## Discussion and conclusion

In the process of species evolution, the size of the cp genome plays an important role (Wang et al. 2008; Tu et al. 2023). In angiosperms, the cp genome size ranges from 120 to 160 kb (Ingvarsson et al. 2003), whereas in plants of the Lamiaceae family, the cp genome size is approximately 150 kb (Yao et al. 2020). This study sequenced and annotated the whole cp genome of *P. virginiana* and compared it with the genomes of plants in the Lamiaceae family. The complete cp genome of *P. virginiana* is 152,880 bp in length, with a total of 131 genes annotated, and the GC content is 38.38%. When compared with the reported Lamiaceae plants such as *Scutellaria tsinyunensis* (152,089 bp, 38.40%) (Shan et al. 2021), *L. ilicifolius* (151,466 bp, 38.60%) (Hou et al. 2024), *S. altaica* (151,779 bp, 38.30%) (Zhao et al. 2020), and *Ajuga forrestii* (150,492 bp, 38.30%) (Tao et al. 2019), the characteristics such as genome size, structure, composition, and GC content are highly similar. This indicates that *P. virginiana* exhibits good conservativeness during the evolutionary process. Previously, studies on the phylogenetic relationships among species of different genera in the Lamiaceae family showed that species of the four genera, *Salvia*, *Lavandula*, *Ocimum*, and *Prunella*, clustered into one branch, while species of the three genera, *Ajuga*, *Scutellaria*, and *Lamium*, clustered into another branch (Han and Zheng 2018; Tao et al. 2019; Li et al. 2019a; Balaji et al. 2021; Hou et al. 2024). However, some research findings also indicated that *Lamium* and *Ocimum* clustered into one branch (Cui et al. 2020). Nevertheless, the results of this study are consistent with the former ones, which further clarify the phylogenetic relationships among the genera of the Lamiaceae family. Meanwhile, it enriches the systematic positions of other genera such as *Physostegia*, *Hanceola*, *Clerodendrum*, *Gomphostemma*, and *Eriophyton* within the Lamiaceae family. The *P. virginiana* characterized in this study is the first complete cp genome released in this genus. *P. virginiana* clustered with *G. lucidum*, *L. ilicifolius*, *L. album*, and *E. wallichii* with a support rate of 100%. These results enrich our understanding of the genetic resources of this genus and provide a molecular basis for the further subfamily classification of the Lamiaceae family. In the future, we will continue to conduct in-depth cp genome sequencing of the Lamiaceae species, and will further refine and improve the classification of Lamiaceae family from the perspectives of tribes and subtribes.

## Supplementary Material

Editing Certificate.pdf

Figure S4.tif

Figure S6.tif

Figure S2.tif

Figure S5.tif

Figure S3.tif

Figure S1.tif

## Data Availability

The data that support the findings of this study are openly available in GenBank of NCBI at https://www.ncbi.nlm.nih.gov/. The complete cp genome has been deposited in GenBank under the accession no. OR832244.2. And the associated Bio-project, SRA, Bio-sample numbers are PRJNA1042873, SRS19602750, and SAMN38323564 respectively.
