# Supplementary figures and images for: Complete chloroplast genome structural and phylogenetic analysis of Physostegia virginiana (L.) Benth. 1930 (Lamiaceae)
Source: Mitochondrial DNA B Resour. 2025 Jul 16;10(8):736–41. doi: 10.1080/23802359.2025.2528568 (PMC12269052; doi:10.1080/23802359.2025.2528568)

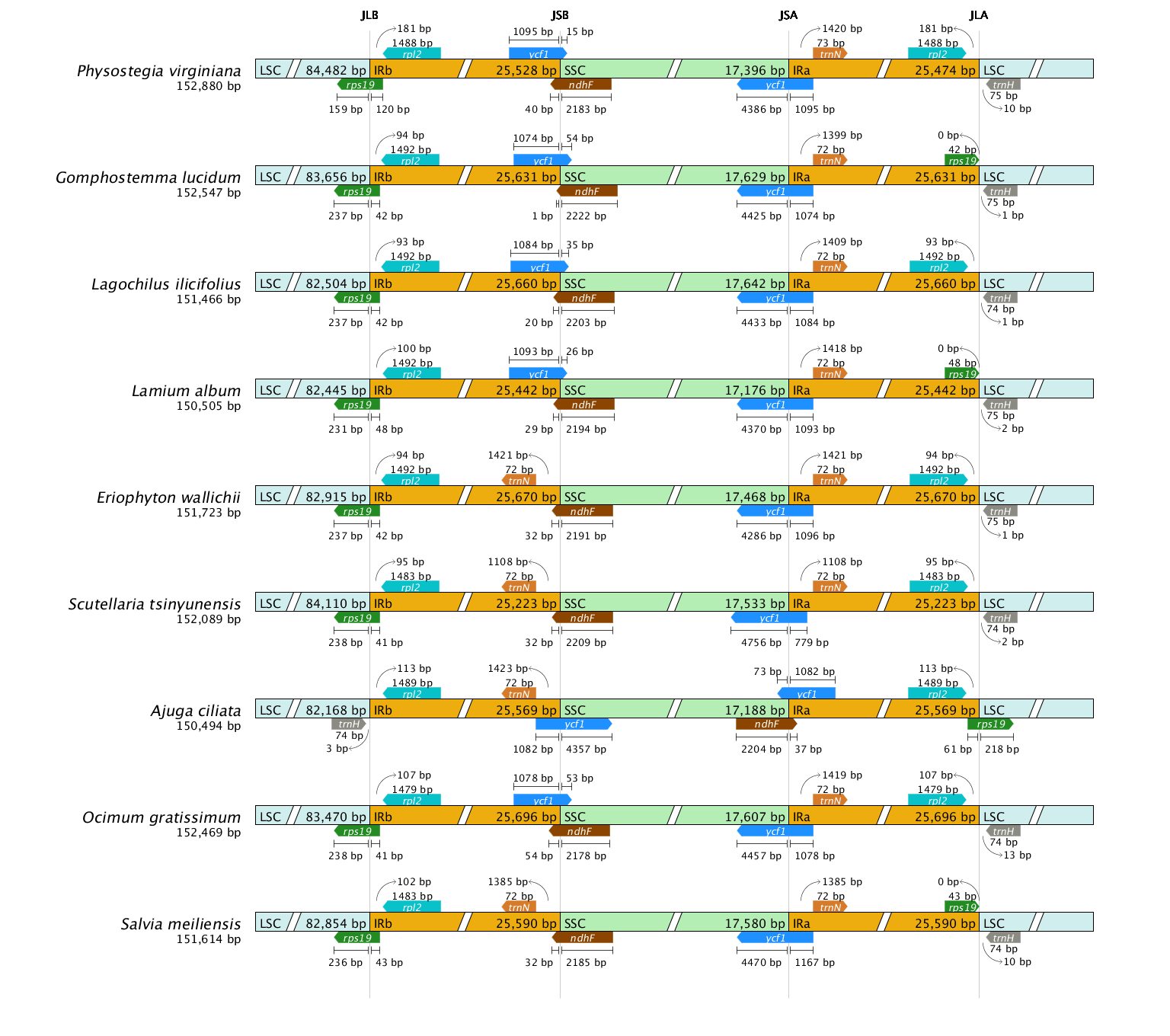

Supplement: Figure S4.tif [file TMDN_A_2528568_SM8506.tif]

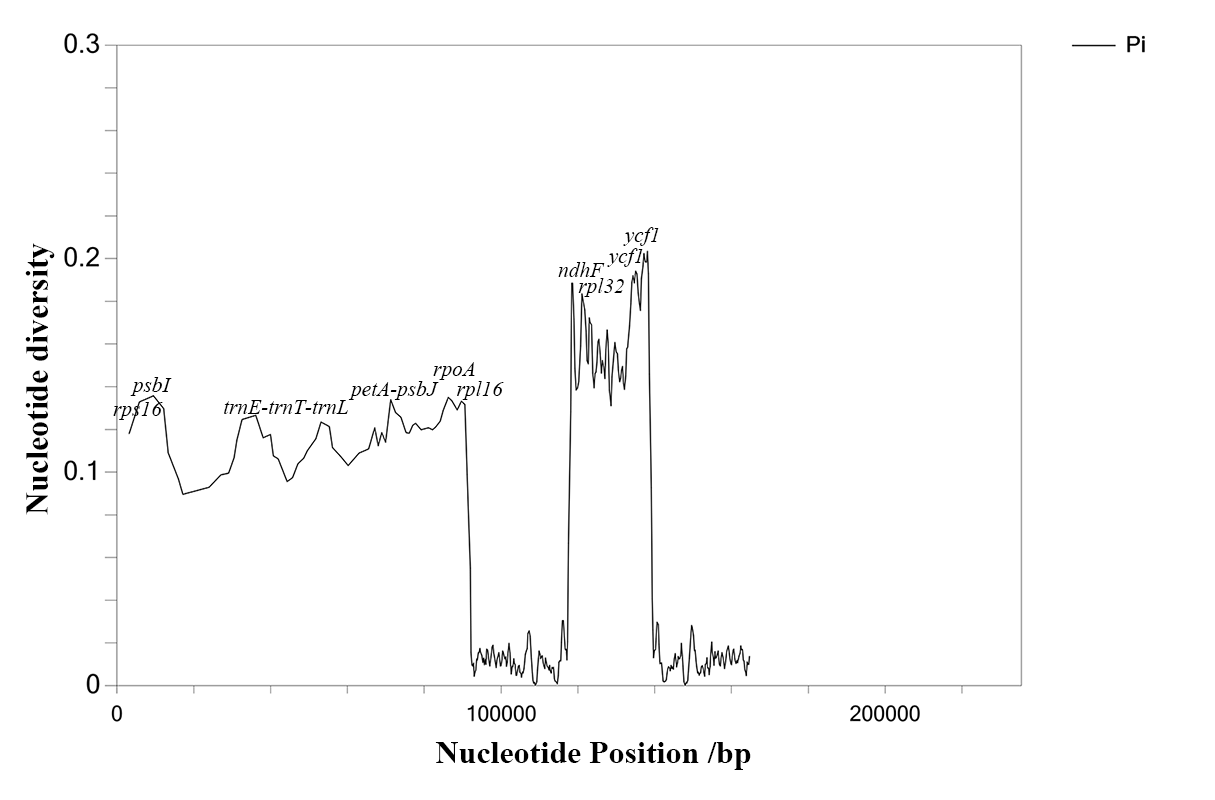

Supplement: Figure S6.tif [file TMDN_A_2528568_SM8505.tif]

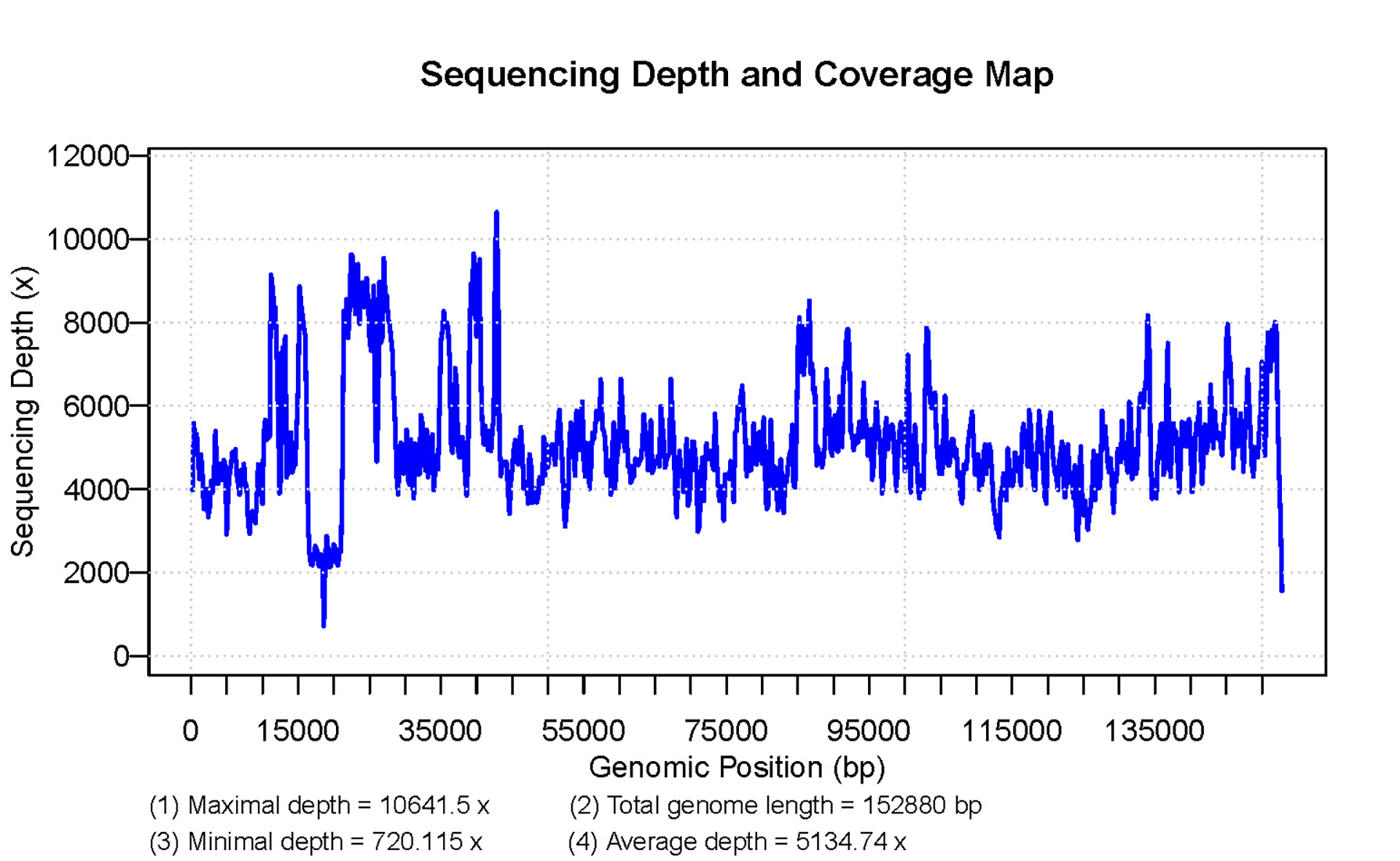

Supplement: Figure S2.tif [file TMDN_A_2528568_SM8504.tif]

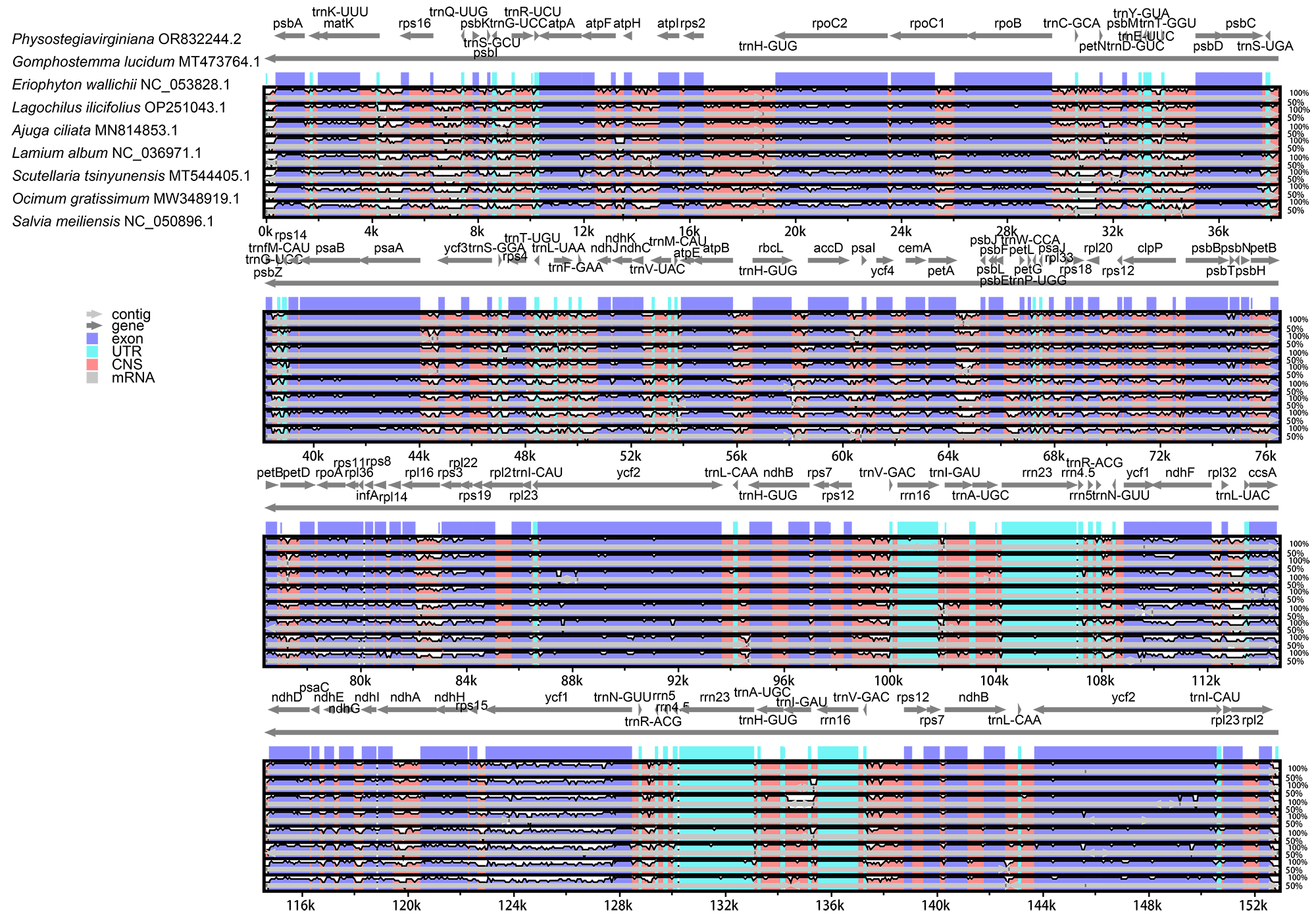

Supplement: Figure S5.tif [file TMDN_A_2528568_SM8503.tif]

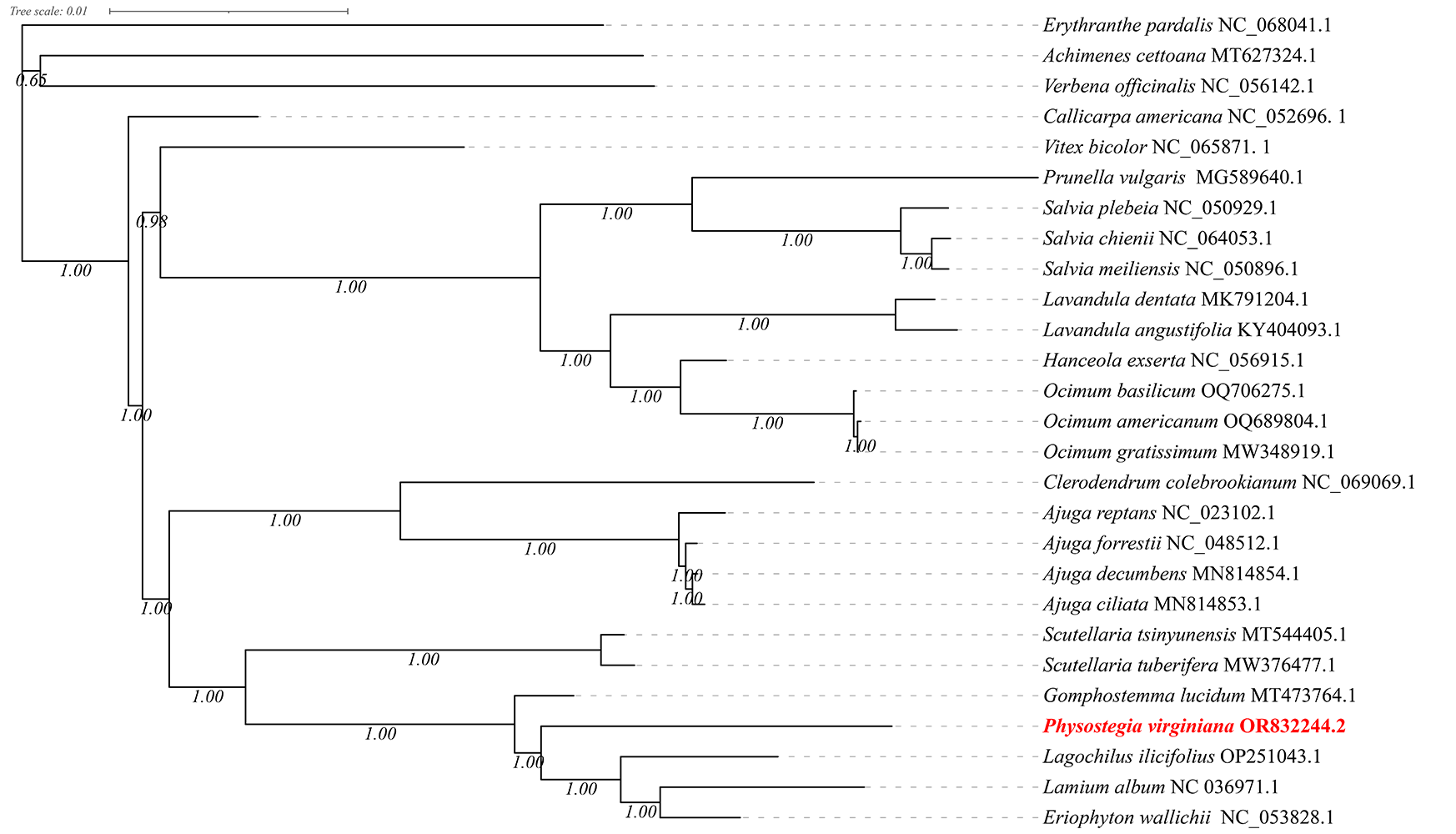

Supplement: Figure S3.tif [file TMDN_A_2528568_SM8502.tif]

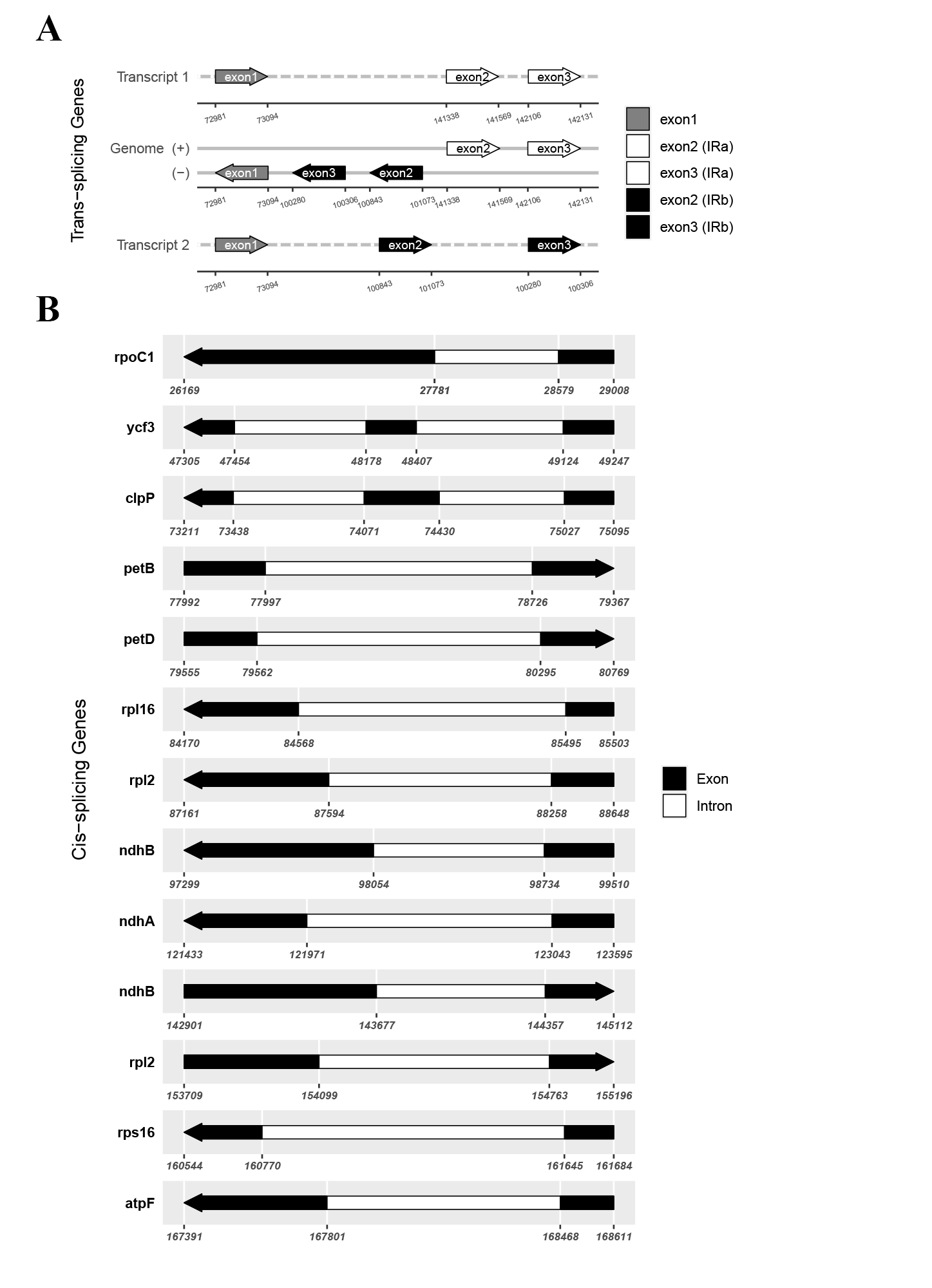

Supplement: Figure S1.tif [file TMDN_A_2528568_SM8501.tif]
